# Digital legal awareness intervention reduces anxiety through enhanced coping strategies: a cluster RCT among Chinese vocational students

**DOI:** 10.3389/fpsyg.2026.1641844

**Published:** 2026-03-04

**Authors:** Han Xiao, Rui Wen, Qiuyan Peng

**Affiliations:** 1 Chongqing Polytechnic University of Electronic Technology, Chongqing, China; 2 Second Affiliated Hospital, Chongqing Medical University, Chongqing, China; 3 Chongqing Education Information Technology and Equipment Center, Chongqing, China

**Keywords:** anxiety reduction, cluster randomized controlled trial, coping strategies, legal education, micro-learning

## Abstract

**Background:**

Traditional legal publicity methods inadequately address both cognitive and psychological dimensions of legal awareness among Chinese vocational college students, who experience heightened anxiety when confronting legal challenges. This study evaluated whether theory-based interactive multimedia legal publicity improves psychological outcomes compared to conventional approaches.

**Methods:**

A cluster randomized controlled trial was conducted with 500 students from 14 classes across 7 colleges at a large vocational college in Southwest China. The intervention group (*n* = 258) received an 8-week interactive multimedia legal publicity program integrating micro-learning principles, narrative psychology, and social cognitive theory through WeChat group discussions and multimedia content delivery. The control group (*n* = 242) received traditional legal publicity through conventional channels. Primary outcomes included anxiety levels (Self-Rating Anxiety Scale) and coping strategies (Simple Coping Style Questionnaire) measured at baseline and post-intervention.

**Results:**

Linear mixed-effects models revealed significant intervention effects. The intervention group showed greater anxiety reduction (group × time interaction: *β* = 2.76, 95% CI [0.7, 4.8]), enhanced positive coping strategies (*β* = −4.92, 95% CI [−6.3, −3.5]), and reduced negative coping patterns (*β* = 3.55, 95% CI [2.4, 4.7]) compared to controls. Effect sizes were moderate to large (Cohen’s *d* = 0.40–0.64). Among students with baseline clinical anxiety, 18.4% in the intervention group versus 11.6% in the control group improved to subclinical levels. Mediation analysis indicated that positive coping strategies completely mediated the intervention-anxiety relationship, accounting for 76.5% of the total effect.

**Conclusion:**

Theory-based interactive multimedia legal publicity may effectively reduce anxiety and enhance adaptive coping among Chinese vocational college students. The intervention’s reliance on familiar platforms and existing institutional structures suggests potential for scalable implementation in Chinese higher education contexts.

## Introduction

Legal publicity represents a cornerstone of China’s rule of law strategy, with particular significance for college students transitioning into full civic participation. Despite government emphasis on legal education, significant challenges persist in both cognitive and psychological dimensions of legal awareness among Chinese college students. Recent studies reveal that approximately 13.7% of Chinese college students experience anxiety symptoms, with vocational college students facing additional pressures related to career preparation and skill development (Chen et al., 2022; Xu et al., 2024). These psychological vulnerabilities are particularly pronounced when students encounter legal challenges, as over 60% report anxiety when confronting legal problems, suggesting a critical need for approaches that address both knowledge acquisition and emotional wellbeing (Yang et al., 2024).

Traditional legal publicity methods have proven inadequate in addressing both cognitive and psychological dimensions of legal awareness, particularly in vocational colleges where students face unique challenges in developing legal literacy alongside professional competencies (Ge et al., 2024). Conventional approaches typically rely on static information dissemination through lectures, printed materials, and one-way communication channels that fail to engage students actively or address their psychological responses to legal challenges (Fu et al., 2024). Moreover, studies of Chinese vocational college students indicate that while 72.9% report no depression symptoms, the prevalence of anxiety-related stress and suboptimal coping strategies suggests room for improvement in mental health support through educational interventions (Xu et al., 2024).

Contemporary digital natives require fundamentally different approaches to information processing and engagement, particularly given the widespread adoption of mobile and social media platforms among Chinese college students (Pan et al., 2024). Interactive multimedia approaches align with established learning theories and offer promising alternatives for legal publicity. Micro-learning theory suggests that breaking complex legal information into digestible, sequential multimedia segments enhances comprehension and reduces cognitive overwhelm, as demonstrated by mobile applications delivering brief intervention modules that significantly improve emotional regulation among university students (Laure et al., 2025). Social cognitive theory emphasizes that observational learning through multimedia case narratives can foster vicarious experiences that build self-efficacy for handling legal challenges, with virtual reality interventions showing effect sizes ranging from 0.344 to 0.492 in reducing anxiety among college students (Man et al., 2024; Kulkarni and Weir, 2024). Research demonstrates that integration of visual, auditory, and interactive elements enhances engagement with complex concepts, while narrative-based content improves emotional processing and reduces anxiety by providing cognitive frameworks for understanding potentially stressful situations (Zulhemay et al., 2024; Bennett and Christensen, 2024). Peer-mediated digital interactions, particularly through platforms like WeChat groups commonly used by Chinese students, create social learning environments that enhance coping strategy development through shared experiences and mutual support (Pan et al., 2024; Luo and Mohammed, 2023).

The theoretical integration of micro-learning, narrative psychology, and social cognitive approaches suggests that digitally-mediated legal publicity can simultaneously address knowledge gaps and psychological wellbeing by creating accessible, emotionally resonant, and socially supportive learning experiences. Empirical evidence supports this integration, with studies demonstrating that multimedia interventions incorporating evidence-based coping strategies, such as the “Atena” chatbot providing audiovisual content and mindfulness techniques, significantly reduce anxiety and stress symptoms among university students (Gabrielli et al., 2021). Similarly, gamified applications like “SuperBetter” that combine cognitive-behavioral strategies with interactive elements show significant improvements in anxiety and depression symptoms compared to control conditions (Bantjes et al., 2024). Chinese college students demonstrate preferences for digital learning platforms that are user-friendly, interactive, and accessible, with mobile learning applications being particularly popular due to their convenience and flexibility (Shi and Zhang, 2024).

However, a significant research gap exists in empirical evidence regarding how digitally-enhanced legal publicity influences psychological outcomes through enhanced coping mechanisms in Chinese educational contexts. While studies have examined knowledge acquisition through innovative approaches and documented the effectiveness of multimedia interventions for mental health, few have rigorously investigated the mediating role of adaptive coping strategies in anxiety reduction within legal publicity contexts. Research on Chinese college students indicates that active and considerate coping styles are associated with better mental health outcomes, while passive and impulsive styles correlate with higher anxiety and depression levels (Yang et al., 2024; Tian et al., 2025). Furthermore, coping strategies have been identified as significant mediators in the relationship between educational interventions and psychological outcomes, yet this mechanism remains unexplored in the context of legal publicity initiatives (Yang et al., 2024).

This study addresses this gap by conducting a cluster randomized controlled trial to evaluate whether a theory-based interactive multimedia legal publicity intervention produces superior psychological outcomes compared to traditional approaches among vocational college students. The intervention integrates micro-learning principles, narrative case presentations, and peer-mediated digital interactions through class-specific WeChat groups and multimedia content delivery to create a comprehensive publicity framework. We hypothesized that this multimedia approach would significantly reduce legal-related anxiety and enhance positive coping strategies through improved information processing, enhanced self-efficacy, and strengthened peer support networks. Furthermore, we predicted that positive coping strategies would mediate the relationship between the intervention and anxiety reduction, providing mechanistic insight into how theory-driven multimedia publicity approaches influence psychological wellbeing among Chinese vocational college students.

Based on the theoretical framework integrating micro-learning principles, narrative psychology, and social cognitive theory, we formulated the following hypotheses:

*H1*: Students receiving the interactive multimedia legal publicity intervention will show significantly greater reductions in anxiety levels compared to students receiving traditional legal publicity (group × time interaction effect).

*H2*: Students receiving the intervention will demonstrate significantly greater improvements in positive coping strategies and greater reductions in negative coping strategies compared to the control group.

*H3*: Positive coping strategies will mediate the relationship between the intervention and anxiety reduction, such that the intervention effect on anxiety operates through enhanced adaptive coping mechanisms.

By examining these psychological mechanisms, this research provides empirical evidence for the theoretical relationship between interactive learning methodologies and psychological wellbeing in legal education contexts, offering practical insights for evidence-based integration of multimedia approaches in legal publicity initiatives within Chinese higher education settings.

## Methods

### Research design

This study employed a cluster randomized controlled trial (CRCT) design to evaluate the psychological impact of interactive multimedia legal publicity on vocational college students. Classes were used as the unit of randomization to minimize contamination between intervention and control groups, following established guidelines for educational intervention research (Yang et al., 2024). The study was conducted at a large vocational college in Southwest China from September 10, 2023 to December 15, 2023. Baseline (T0) measurements were collected 1 week before the intervention (September 3–7, 2023), and post-intervention (T1) measurements were obtained 1 week after the completion of the 8-week intervention period (December 18–22, 2023).

The studies involving human participants were reviewed and approved by the Academic Committee of Chongqing Polytechnic University of Electronic Technology (Approval Number: CQUET-EC-2023-001). The study was conducted in accordance with the Declaration of Helsinki and the ethical standards of the institutional research committee. Written informed consent was obtained from all participants prior to enrollment in the study. Participants were informed of their right to withdraw at any time without penalty. All data were anonymized during collection and analysis to protect participant privacy and confidentiality.

### Participants and sampling

Participants were recruited from 14 classes across 7 academic departments within a large vocational college in Southwest China. Eligible participants were full-time students aged 18–25 years who provided informed consent and had active WeChat accounts for participation in class group communications. Students who had received systematic legal publicity activities within the previous 3 months or who had a diagnosed psychological disorder requiring clinical treatment were excluded from the study.

Using stratified random sampling, classes were first stratified by college department to ensure representation across academic disciplines. Within each stratum, classes were randomly assigned to intervention or control conditions using a computer-generated random number sequence created by a researcher not involved in participant recruitment or assessment. The randomization was performed at the class level rather than the individual level to minimize contamination between conditions, as students within the same class share common WeChat groups and daily interactions. Allocation concealment was maintained by using sequentially numbered, opaque, sealed envelopes that were opened only after all classes within each stratum had been enrolled. Class advisors and students were informed of their group assignment only after baseline data collection was completed.

Based on power analysis using G*Power software (version 3.1.9.6; *α* = 0.05, power = 0.90, expected effect size *d* = 0.50 derived from similar multimedia intervention studies), and accounting for the design effect of cluster randomization (DEFF = 1 + *δ*(m−1), where δ represents the assumed intraclass correlation coefficient of 0.05, consistent with typical values reported in educational intervention research (Hedges and Hedberg, 2007), and m represents the average cluster size of 36 students per class), the required sample size was determined to be 154 students per group. To accommodate potential attrition (estimated at 10%–20% based on similar educational intervention studies), 14 classes with approximately 500 students were recruited. The final sample comprised 500 students with 258 in the intervention group and 242 in the control group.

### Intervention

#### Theory-based interactive multimedia legal publicity program

The intervention group consisted of 7 classes that participated in an 8-week interactive multimedia legal publicity campaign designed to enhance legal awareness while reducing law-related anxiety. The program was grounded in micro-learning theory, narrative psychology, and social cognitive theory, and implemented through class-specific digital channels to ensure precise targeting and avoid contamination of control classes.

The intervention utilized a progressive micro-content delivery approach based on micro-learning theory principles that segment complex information into manageable units to reduce cognitive load (Laure et al., 2025). Content was delivered through class WeChat groups with bi-weekly posts featuring 3–5 min legal education micro-videos addressing common vocational student legal concerns across four thematic modules: campus loan risks during weeks one and two, online fraud prevention during weeks three and four, employment rights protection during weeks five and six, and rental dispute resolution during weeks seven and eight. Each micro-video followed a structured four-segment approach encompassing problem identification through relatable scenarios, case narrative presented in first-person perspective to facilitate identification and emotional engagement, solution pathway with specific legal procedures and rights protection steps, and reflection prompt to encourage personal application. Visual infographics illustrated specific legal procedures and rights protection pathways, with content professionally produced using smartphone recording and basic editing software to ensure accessibility and cost-effectiveness.

The intervention incorporated structured peer interactions within class WeChat groups to promote observational learning and coping strategy development based on social cognitive theory (Zulhemay et al., 2024). Following each content delivery, students engaged in guided discussions facilitated by class advisors, with prompts designed to encourage reflection and collaborative problem-solving. Monthly case discussion sessions encouraged students to share relevant experiences and collectively generate solution strategies, fostering social support and vicarious learning experiences. Gamified elements were incorporated to enhance engagement, including weekly legal knowledge mini-quizzes delivered through WeChat group functions, monthly knowledge competitions with small incentives, and interactive polls addressing common legal misconceptions. These activities provided immediate feedback and reinforcement, supporting self-efficacy development through mastery experiences.

To ensure consistent implementation across intervention classes, several fidelity monitoring procedures were employed. All class advisors received a standardized 2-h orientation session on facilitation techniques and intervention protocols prior to the study. Class advisors completed weekly implementation checklists documenting content delivery and discussion facilitation, with high overall adherence observed across all intervention classes. Research assistants conducted bi-weekly reviews of WeChat group activity logs to verify content delivery timing and student engagement levels. The research team maintained regular communication with class advisors throughout the intervention period to address implementation challenges and ensure protocol adherence.

#### Control group management

The control group consisted of seven classes that received traditional legal publicity through conventional channels of equivalent frequency and duration. This included printed informational brochures distributed bi-weekly, traditional classroom-based presentations covering identical legal topics, and static informational displays in common areas. Control classes were managed by different advisors and maintained complete separation from intervention activities to prevent contamination. All intervention materials were provided to control group participants after completion of data collection as an ethical consideration.

### Measures

Data were collected using validated Chinese instruments at baseline (T0) and post-intervention (T1), selected based on their established psychometric properties in Chinese college student populations.

Originally developed by Zung (1971) and validated in Chinese contexts, the SAS assesses anxiety symptoms through 20 items rated on a 4-point scale (1 = none or a little of the time, 4 = most or all of the time). Sample items include “I feel more nervous and anxious than usual” and “I feel afraid for no reason at all.” Items 5, 9, 13, 17, and 19 were reverse-scored before analysis. Raw scores were multiplied by 1.25 to obtain standard scores, with scores ≥50 indicating clinical anxiety levels. The Chinese version demonstrates good internal consistency in college student populations, with previous studies reporting Cronbach’s *α* coefficients ranging from 0.82 to 0.89 (Yang et al., 2024). Confirmatory factor analysis supports a two-factor structure distinguishing cognitive and somatic anxiety symptoms. In the current study, internal consistency was adequate at both time points (T0: *α* = 0.78; T1: *α* = 0.76).

Developed by Xie (1998) specifically for Chinese populations, the SCSQ measures coping strategies through 20 items divided into positive coping and negative coping subscales, each rated on a 4-point scale (0 = never use, 3 = frequently use). The positive coping subscale (14 items) includes problem-solving, seeking social support, and cognitive reframing strategies, with sample items such as “Try to see the positive side of things” and “Seek help from professionals.” The negative coping subscale (6 items) encompasses avoidance, denial, and potentially maladaptive strategies, including items like “Fantasize how much better it would be if things were different” and “Make yourself feel better through smoking, drinking, taking medicine, or eating.” Higher scores indicate greater utilization of the respective coping style. The original validation study reported acceptable internal consistency for both subscales (positive coping: *α* = 0.78; negative coping: *α* = 0.72), and subsequent studies in Chinese college samples have demonstrated similar reliability patterns (Xu et al., 2024). Factor analysis confirms the two-factor structure with clear distinction between adaptive and maladaptive coping strategies. In the present study, reliability was acceptable for the positive coping subscale (T0: *α* = 0.67; T1: *α* = 0.73), while the negative coping subscale showed lower internal consistency consistent with previous findings in similar populations.

A structured questionnaire collected information on age, gender, ethnicity, academic major, academic performance ranking (categorized as top 25%, 25%–50%, 50%–75%, or bottom 25%), residential background (urban vs. rural), and paternal education level (ranging from no formal education to doctoral degree) to characterize the sample and identify potential confounding variables.

Prior to main analyses, the factor structure of all instruments was examined in the current sample. Both the SAS and SCSQ demonstrated factor structures consistent with their established theoretical models and previous validation studies in Chinese college student populations. The SAS supported the two-factor structure distinguishing cognitive and somatic anxiety components, while the SCSQ confirmed the distinction between positive and negative coping strategies. Measurement invariance across intervention and control groups was established for both instruments, supporting the validity of between-group comparisons.

### Data collection and analysis

Data collection occurred in standardized classroom environments under supervision of trained research assistants, with class teachers absent to minimize response bias. Participants received identical instructions, with approximately 30 min allocated for questionnaire completion. Participant anonymity was maintained through coded identification systems.

Data analysis was conducted using R (version 4.1.0). Data preprocessing included multiple imputation for missing values (<5%) and outlier detection. Normality was assessed via Shapiro–Wilk tests. Baseline group comparisons utilized chi-square tests for categorical variables and Mann–Whitney *U* tests for continuous variables.

Primary analyses used linear mixed-effects models (lme4 package) with class-level clustering as a random effect. Fixed effects included group, time, group × time interaction, and covariates (gender, academic ranking, residence). The group × time interaction served as the primary parameter of interest. Moderation analyses examined three-way interactions for gender, major type, and residence.

Clinical significance was evaluated by calculating improvement rates for participants with baseline clinical anxiety (SAS ≥ 50) and associated risk ratios. Effect sizes were calculated using Cohen’s *d*. Mediation analysis employed the mediation package with 5,000 bootstrap resamples to examine whether positive coping mediated the intervention-anxiety relationship.

All tests were two-sided with *α* = 0.05. Bonferroni corrections were applied for multiple comparisons. Missing data were addressed through multiple imputation when appropriate.

## Results

### Participant characteristics and baseline assessment

A total of 500 students from 14 classes across 7 academic departments at a large vocational college in Southwest China. The intervention group comprised 258 students from 7 classes, while the control group included 242 students from 7 classes. Table 1 presents the baseline characteristics of both groups.

**Table 1:** Baseline characteristics of study participants.

Variable	Intervention	Control	*p*-value
	(*n* = 258)	(*n* = 242)	
Gender			
Male	145 (56.2)	138 (57.0)	0.924
Female	113 (43.8)	104 (43.0)	
Ethnicity			
Han	245 (95.0)	230 (95.0)	1
Minority	13 (5.0)	12 (5.0)	
Academic ranking			
Bottom 25%	67 (26.0)	60 (24.8)	0.27
50%–75%	53 (20.5)	62 (25.6)	
25%–50%	78 (30.2)	57 (23.6)	
Top 25%	60 (23.3)	63 (26.0)	
Residence			
Urban	138 (53.5)	94 (38.8)	0.001
Rural	120 (46.5)	148 (61.2)	
Family education background			
No education	6 (2.3)	2 (0.8)	0.208
Primary school	51 (19.8)	37 (15.3)	
Middle school	93 (36.0)	100 (41.3)	
High school	72 (27.9)	68 (28.1)	
Technical school	8 (3.1)	14 (5.8)	
College diploma	12 (4.7)	13 (5.4)	
Bachelor’s degree	16 (6.2)	8 (3.3)	
SAS^a^, median [IQR]	56.00 [50.00, 64.00]	56.00 [49.25, 65.00]	0.79
SCSQ+^b^ (positive coping), median [IQR]	23.00 [19.00, 28.00]	23.00 [19.00, 27.00]	0.813
SCSQ−^c^ (negative coping), median [IQR]	14.00 [10.00, 17.00]	14.00 [11.00, 17.75]	0.879

SAS, self-rating anxiety scale; SCSQ+, positive coping subscale; SCSQ−, negative coping subscale; IQR, interquartile range. a = SAS (Self-Rating Anxiety Scale); b = SCSQ+ (Positive coping subscale); c = SCSQ- (Negative coping subscale).

The groups were comparable in most demographic variables, including gender (intervention: 56.2% male; control: 57.0% male; *χ*^2^(1) = 0.01, *p* = 0.924), ethnicity (both groups: 95.0% Han; *χ*^2^(1) = 0.00, *p* = 1.000), academic ranking (*χ*^2^(3) = 3.92, *p* = 0.270), and Family education background (based on the highest education level of either parent) level (*χ*^2^(6) = 8.44, *p* = 0.208). However, a significant difference was observed in residence type, with a higher proportion of urban residents in the intervention group (53.5%) compared to the control group (38.8%; *χ*^2^(1) = 10.69, *p* = 0.001). To account for this difference, we included residence as a covariate in all primary analyses and conducted sensitivity analyses with and without this covariate. The inclusion of residence did not substantially alter the pattern or significance of results, suggesting that the observed intervention effects were robust to this baseline difference.

At baseline, no significant differences were found in anxiety levels (SAS) (intervention median = 56.00, IQR = [50.00, 64.00]; control median = 56.00, IQR = [49.25, 65.00]; U = 30672.00, *p* = 0.790), positive coping strategies (SCSQ+) (intervention median = 23.00, IQR = [19.00, 28.00]; control median = 23.00, IQR = [19.00, 27.00]; U = 30884.00, *p* = 0.813), and negative coping strategies (SCSQ−) (intervention median = 14.00, IQR = [10.00, 17.00]; control median = 14.00, IQR = [11.00, 17.75]; U = 30950.50, *p* = 0.879). These results indicate that the intervention and control groups were well-balanced in terms of psychological outcomes at baseline.

### Main intervention effects

Prior to hypothesis testing, we examined the intraclass correlation coefficients (ICCs) to assess the degree of clustering within classes and justify the use of mixed-effects models. The ICCs for the primary outcome variables were as follows: anxiety levels showed an ICC of 0.048, positive coping strategies showed an ICC of 0.052, and social support showed an ICC of 0.061. These values indicate that approximately 5%–6% of the variance in outcomes was attributable to class-level differences, confirming the appropriateness of multilevel modeling to account for the nested data structure. These ICC values, combined with the cluster sizes, yielded design effects greater than 1.5, further supporting the decision to use class as the unit of randomization and to include random intercepts for class in all analyses.

The mixed linear model analysis controlling for class-level clustering (Table 2) revealed significant intervention effects across all psychological outcomes, providing support for Hypotheses 1 and 2. Both groups showed improvements over time, but the intervention group demonstrated significantly better outcomes compared to the control group.

**Table 2:** Linear mixed-effects model analysis of intervention effects.

Predictor	Anxiety (SAS)		Positive coping (SCSQ+)		Negative coping (SCSQ-)	
	*β*	95% CI	*β*	95% CI	*β*	95% CI
Intervention	Reference		Reference		Reference	
Control	−0.08	−1.7, 1.6	−0.18	−2.2, 1.9	0.1	−2.0, 2.2
Baseline	Reference		Reference		Reference	
Post-intervention	−3.39***	−4.8, −2.0	5.41***	4.4, 6.4	−3.99***	−4.8, −3.2
Male	Reference		Reference		Reference	
Female	0.8	−0.3, 1.9	−0.08	−0.9, 0.7	0.23	−0.4, 0.9
Linear trend	1.35*	0.3, 2.4	−0.80*	−1.5, −0.1	0.06	−0.5, 0.6
Quadratic trend	−0.06	−1.1, 1.0	−0.67	−1.4, 0.0	0.02	−0.5, 0.6
Cubic trend	−0.54	−1.6, 0.5	0.54	−0.2, 1.3	0.21	−0.4, 0.8
Control × post-intervention	2.76**	0.7, 4.8	−4.92***	−6.3, −3.5	3.55***	2.4, 4.7

*p < 0.05, **p < 0.01, ***p < 0.001. CI, confidence interval.

For anxiety levels (SAS), both groups showed reduced anxiety at post-intervention (*β* = −3.39, 95% CI [−4.8, −2.0]), but this improvement was significantly attenuated in the control group as indicated by the significant group × time interaction (*β* = 2.76, 95% CI [0.7, 4.8]). This result supports Hypothesis 1, suggesting that the interactive multimedia legal education was more effective in reducing anxiety compared to traditional legal education.

For positive coping strategies (SCSQ+), there was an overall improvement over time (*β* = 5.41, 95% CI [4.4, 6.4]), with a significant group × time interaction (*β* = −4.92, 95% CI [−6.3, −3.5]) indicating that the intervention group showed greater improvement in positive coping than the control group. For negative coping strategies (SCSQ−), post-intervention scores were lower overall (*β* = −3.99, 95% CI [−4.8, −3.2]), with a significant group × time interaction (*β* = 3.55, 95% CI [2.4, 4.7]), showing that the reduction in negative coping was more pronounced in the intervention group. These findings support Hypothesis 2.

Academic ranking showed a significant effect on some outcomes, with students in the top 25% showing higher anxiety levels (*β* = 1.35, *p* < 0.05, 95% CI [0.3, 2.4]) and lower positive coping (*β* = −0.80, *p* < 0.05, 95% CI [−1.5, −0.1]) compared to those in other ranking categories.

### Moderation effects

The moderation analysis (Table 3) examined whether the intervention effects varied by gender or urban–rural residence through three-way interactions (group × time × moderator). Contrary to our exploratory expectations, none of the moderation effects reached statistical significance.

**Table 3:** Moderation effects analysis.

Moderator	Outcome variable	Estimatea	SE	*t*-value	*p*-value
Gender (male vs. female)	Anxiety (SAS)	−0.056	2.108	−0.027	0.979
	Positive coping (SCSQ+)	0.299	1.433	0.209	0.835
	Social support (SSRS)	0.595	2.151	0.276	0.782
Residence (urban vs. rural)	Anxiety (SAS)	−0.019	2.113	−0.009	0.993
	Positive coping (SCSQ+)	0.228	1.437	0.159	0.874
	Social support (SSRS)	0.233	2.165	0.107	0.915

Table shows coefficients for three-way interactions (Group × Time × Moderator) from linear mixed-effects models with class as random intercept. None of the interaction effects reached statistical significance (all *p* > 0.05), indicating that intervention effects did not differ significantly across demographic subgroups.

For anxiety levels, the three-way interaction with gender was not significant (*β* = −0.056, SE = 2.108, *t* = −0.027, *p* = 0.979), nor was the interaction with residence (*β* = −0.019, SE = 2.113, *t* = −0.009, *p* = 0.993). Similarly, for positive coping strategies, neither gender (*β* = 0.299, SE = 1.433, *t* = 0.209, *p* = 0.835) nor residence (*β* = 0.228, SE = 1.437, *t* = 0.159, *p* = 0.874) significantly moderated the intervention effect. The same pattern was observed for social support outcomes, with non-significant interactions for both gender (*β* = 0.595, SE = 2.151, *t* = 0.276, *p* = 0.782) and residence (*β* = 0.233, SE = 2.165, *t* = 0.107, *p* = 0.915).

These null findings suggest that the intervention was equally effective across gender and residential background subgroups. Rather than indicating a limitation, this pattern supports the broad applicability of the interactive multimedia legal publicity approach across diverse student populations within the vocational college context.

### Clinical significance and mediation analysis

Analysis of clinical outcomes revealed that among students with clinical anxiety at baseline (SAS ≥ 50), the improvement rate to subclinical levels was higher in the intervention group (18.4%) compared to the control group (11.6%). The intervention yielded a risk ratio of 1.59 and a number needed to treat of 14.7, indicating that approximately 15 students need to receive the intervention for one additional student to show clinically meaningful improvement.

**Figure 1:**
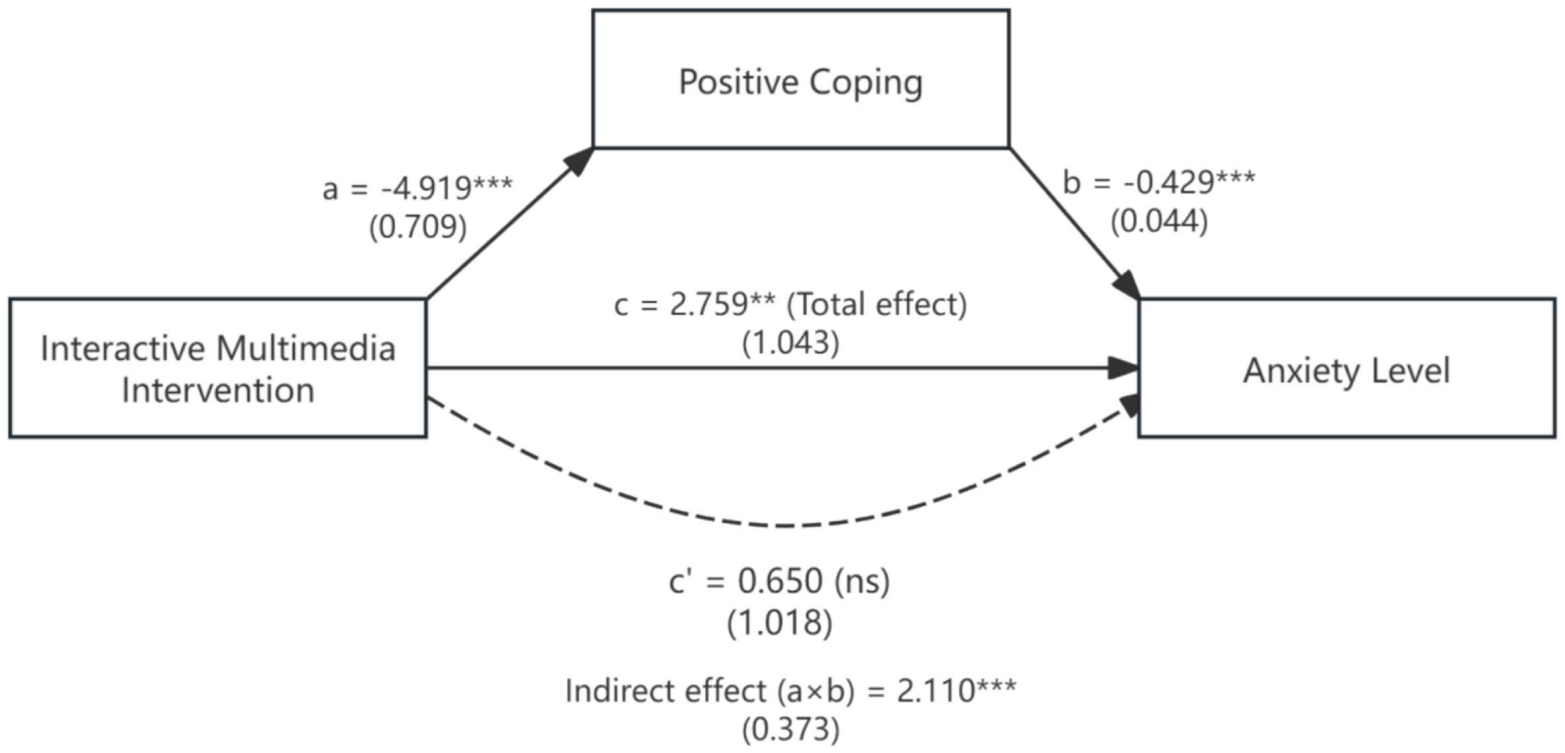
Mediation model of intervention effects on anxiety through positive coping strategies. Unstandardized coefficients are presented with standard errors in parentheses. Path a represents the effect of intervention on positive coping; path b represents the effect of positive coping on anxiety; path c represents the total effect; path c′ represents the direct effect after controlling for the mediator. The indirect effect (a × b = 2.110) was significant (*p* < 0.001), and the proportion mediated was 76.5%. Complete mediation was supported as the direct effect (c′) became non-significant after including the mediator. ***p < 0.001, **p < 0.01, ns, not significant.

Mediation analysis examined whether positive coping strategies mediated the relationship between the intervention and anxiety reduction, providing a test of Hypothesis 3 (Figure 1). Following Baron and Kenny’s approach with bootstrap confidence intervals, we tested the indirect effect of the intervention on anxiety through positive coping strategies.

The results supported complete mediation. The intervention had a significant effect on positive coping (path a: *β* = −4.919, SE = 0.709, *p* < 0.001), indicating that participants in the intervention group showed substantially greater improvements in positive coping strategies compared to controls. Positive coping, in turn, significantly predicted anxiety levels (path b: *β* = −0.429, SE = 0.044, *p* < 0.001), with higher positive coping associated with lower anxiety. The total effect of the intervention on anxiety was significant (path c: *β* = 2.759, SE = 1.043, *p* = 0.008), confirming the overall effectiveness of the intervention in reducing anxiety.

Critically, after controlling for positive coping, the direct effect of the intervention on anxiety became non-significant (path c′: *β* = 0.650, SE = 1.018, *p* = 0.524). The indirect effect through positive coping was significant (a × b = 2.110, bootstrap 95% CI [1.42, 2.85], *p* < 0.001), with the proportion of the total effect mediated being 76.5%. According to Baron and Kenny’s criteria, when the direct effect becomes non-significant while the indirect effect remains significant, this constitutes complete mediation.

These findings strongly support Hypothesis 3, indicating that the intervention’s anxiety-reducing effect operates primarily through enhanced positive coping strategies. These findings suggest that interactive multimedia legal publicity reduces anxiety by enhancing students’ positive coping strategies rather than through direct effects on anxiety per se. This mechanism highlights the importance of targeting coping skill development in legal publicity initiatives rather than focusing exclusively on knowledge acquisition.

## Discussion

This study provides compelling evidence that theory-based interactive multimedia legal publicity significantly improves psychological outcomes among Chinese vocational college students compared to traditional approaches. Our findings reveal substantial positive effects across multiple psychological dimensions, establishing clear advantages for interactive multimedia approaches in reducing anxiety, enhancing positive coping strategies, and decreasing maladaptive coping mechanisms. Most importantly, this research elucidates the psychological mechanisms underlying these benefits, demonstrating that positive coping strategies completely mediate the relationship between multimedia interventions and anxiety reduction.

### Main intervention effects and theoretical implications

The significant reduction in anxiety levels observed in the intervention group (Cohen’s *d* = 0.64) represents a robust effect that exceeds typical outcomes reported in educational anxiety interventions and approaches the effectiveness of specialized clinical interventions. Our results substantially surpass the average effect sizes found in virtual reality-based interventions for college student anxiety (*d* = 0.344–0.492) documented by Man et al. (2024) and Kulkarni and Weir (2024), while remaining within the range of credible psychological interventions. This enhanced effectiveness may be attributed to our theory-driven integration of micro-learning, narrative psychology, and social cognitive approaches, which simultaneously addresses cognitive load reduction, emotional engagement, and social learning processes.

The micro-learning component of our intervention, delivering content in 3–5 min segments, appears to have effectively reduced cognitive overwhelm that often accompanies legal information processing. This aligns with theoretical predictions that breaking complex information into manageable units enhances comprehension while reducing anxiety-provoking cognitive load (Laure et al., 2025). The narrative-based presentation of legal cases through first-person storytelling likely facilitated emotional connection and identification, helping students develop cognitive frameworks for understanding and managing legal stressors. This finding supports narrative psychology principles suggesting that story-based learning enhances emotional processing and reduces uncertainty-related anxiety (Zulhemay et al., 2024).

The substantial improvement in positive coping strategies (Cohen’s *d* = 0.52) and reduction in negative coping patterns (Cohen’s *d* = 0.40) demonstrate that the intervention successfully enhanced students’ psychological resources for managing legal challenges. These improvements align with social cognitive theory predictions that observational learning through peer interactions and multimedia modeling can enhance self-efficacy and promote adaptive coping responses (Pan et al., 2024). The WeChat group discussions and peer sharing components likely provided vicarious learning experiences that expanded students’ repertoire of coping strategies while reducing their reliance on avoidance and other maladaptive responses.

### Mediation analysis and psychological mechanisms

Our mediation analysis provides critical insight into the psychological mechanisms underlying the intervention’s effectiveness. The finding that positive coping strategies completely mediated the relationship between the intervention and anxiety reduction (76.5% of total effect) reveals the central role of enhanced psychological resources in promoting mental health. This result extends beyond previous research by demonstrating not merely that multimedia interventions reduce anxiety, but how they accomplish this effect through enhanced adaptive coping capabilities.

This complete mediation pattern aligns with theoretical frameworks emphasizing the importance of coping resources in psychological adjustment. The intervention’s multiple components likely contributed synergistically to coping enhancement: micro-learning modules provided structured knowledge that enhanced problem-solving confidence, narrative case presentations offered emotional frameworks for understanding legal stressors, and peer discussions facilitated social learning of diverse coping strategies. The WeChat group environment, familiar and comfortable for Chinese college students, created a supportive social context that encouraged coping skill practice and mutual support (Luo and Mohammed, 2023).

The complete mediation finding carries important theoretical and practical implications. The non-significant direct effect after controlling for positive coping indicates that the intervention’s anxiety-reducing benefit operates primarily through enhanced adaptive coping capabilities rather than through alternative pathways such as direct knowledge acquisition or reduced legal uncertainty. This pattern suggests that coping skill development represents the critical mechanism through which multimedia legal publicity promotes psychological wellbeing among vocational students. From a practical perspective, these findings underscore the importance of explicitly incorporating coping strategy training into legal education programs, as knowledge transmission alone may be insufficient to achieve meaningful reductions in law-related anxiety.

### Moderation effects and individual differences

Contrary to our exploratory expectations, the moderation analyses revealed no significant three-way interactions between group, time, and demographic variables including gender and residential background. The intervention effects on anxiety reduction, positive coping enhancement, and social support did not differ significantly across these subgroups. While this null finding might initially appear disappointing, it actually carries important practical implications for intervention implementation.

The absence of significant moderation effects suggests that the interactive multimedia legal publicity intervention was equally effective for male and female students, as well as for students from urban and rural backgrounds. This broad applicability is a strength rather than a limitation, as it indicates that the intervention can be implemented across diverse student populations without requiring tailored modifications for specific demographic groups (Cuijpers et al., 2016). From a practical standpoint, this finding simplifies implementation decisions and suggests that educational institutions can deploy the intervention universally rather than targeting specific subpopulations.

Several factors may explain why the intervention demonstrated consistent effectiveness across demographic subgroups. First, the digital delivery platform through WeChat is ubiquitous among Chinese college students regardless of gender or residential background, ensuring equal access and familiarity with the intervention medium (Pan et al., 2024). Second, the legal topics addressed in the intervention, including campus loans, online fraud, employment rights, and rental disputes, represent universal concerns facing vocational college students irrespective of their demographic characteristics. Third, the combination of multiple theoretical approaches including micro-learning, narrative psychology, and social cognitive theory may have created a sufficiently comprehensive intervention that resonated with diverse learning preferences and needs (Laure et al., 2025).

These findings contrast with some previous research suggesting differential responses to educational interventions based on gender or socioeconomic background. While gender differences in psychological processes have been documented across various domains (Hyde, 2014), such differences may be attenuated when interventions are designed with universal accessibility and relevance in mind. Our results align with studies demonstrating that well-designed digital interventions can achieve equitable outcomes across diverse populations when the content is universally relevant and the delivery platform is equally accessible. A systematic review of digital mental health interventions for college students found that technology-based approaches can effectively reach diverse student populations and produce comparable outcomes across demographic groups (Lattie et al., 2019).

### Cultural and contextual considerations

The intervention’s effectiveness in the Chinese vocational college context reflects important cultural and educational factors that enhance its relevance and impact. Traditional Chinese educational approaches, influenced by Confucian-heritage pedagogical traditions, have historically emphasized knowledge transmission through direct instruction and memorization (Yang et al., 2024). Our intervention’s success suggests that contemporary Chinese students, particularly digital natives, benefit from more interactive and socially-mediated learning approaches that complement rather than replace traditional methods.

The prominence of WeChat in Chinese digital culture provided an ideal platform for intervention delivery, as students were already comfortable with and actively engaged in class group communications (Pan et al., 2024). This cultural familiarity likely enhanced intervention engagement and reduced barriers to participation that might exist with less familiar platforms or technologies. The integration of peer discussion and collective problem-solving also aligns with Chinese cultural values emphasizing group harmony and mutual support in learning contexts.

The specific legal topics addressed in the intervention—campus loans, online fraud, employment rights, and rental disputes—reflect the most pressing concerns facing Chinese vocational college students as they transition toward independent adult roles. By addressing these authentic, personally relevant challenges, the intervention achieved both educational and psychological objectives that resonate with students’ immediate needs and long-term career preparation goals.

The intervention’s effectiveness in the Chinese vocational college context reflects important cultural and educational factors that merit consideration for understanding the underlying mechanisms and potential generalizability (Yang et al., 2024). Chinese cultural values and communication patterns likely influenced both the intervention design and participant responses in several important ways.

The collectivist orientation prevalent in Chinese culture may have enhanced the effectiveness of the peer-mediated learning components. Hofstede’s cultural dimensions framework identifies China as a highly collectivist society where group harmony, interdependence, and social relationships take precedence over individual autonomy (Hofstede, 2011). The WeChat group discussions fostered a sense of shared experience and mutual support that aligns with Chinese cultural emphases on group harmony and collective problem-solving (Luo and Mohammed, 2023). Students may have been more willing to engage in discussions and share experiences within the familiar context of their class WeChat groups, where established relationships and social norms facilitated open communication about potentially sensitive legal concerns.

Confucian educational traditions emphasizing respect for knowledge and authority may have facilitated acceptance of legal information delivered through institutional channels. Li’s (2012) comparative analysis of Eastern and Western learning models highlights how Chinese learners are socialized to value knowledge acquisition as a moral endeavor and to respect teachers and educational institutions as sources of wisdom. The intervention’s integration with existing class structures and delivery by class advisors positioned the content within established educational hierarchies that Chinese students are accustomed to respecting. This alignment with Confucian educational values, which emphasize hierarchical relationships between teachers and students alongside the moral purposes of education (Tan, 2017), may have enhanced the perceived credibility and relevance of the legal information provided.

The widespread adoption of WeChat as a primary communication platform among Chinese young adults provided a familiar and comfortable medium for intervention delivery (Pan et al., 2024). Unlike interventions requiring adoption of new technologies, our approach leveraged existing digital habits and social networks, reducing barriers to engagement. The integration of multimedia content with social interaction features aligned with Chinese students’ established patterns of digital communication and information consumption (Shi and Zhang, 2024).

These cultural considerations suggest that while the specific intervention components demonstrated effectiveness in the Chinese context, adaptation may be necessary for implementation in cultures with different communication norms, educational traditions, or digital platform preferences. A comprehensive review of meta-analyses on culturally adapted mental health interventions confirms that cultural adaptation of evidence-based interventions produces moderate to large effect sizes, and that interventions should be tailored to specific cultural contexts to maximize engagement and outcomes (Rathod et al., 2018). Future research should examine whether similar interventions produce comparable effects in other cultural contexts and identify which intervention components are culturally universal versus culturally specific.

### Limitations and future research directions

Several methodological limitations warrant consideration in interpreting these findings. First, the single-institution design limits generalizability across China’s diverse vocational education landscape, as different institutional cultures and regional characteristics may influence intervention effectiveness. Second, the immediate post-intervention assessment precludes evaluation of long-term effects, particularly regarding coping skill maintenance and legal knowledge retention. Third, the reliance on self-report measures introduces potential social desirability biases, though this is mitigated by the use of validated instruments and anonymous data collection.

Future research should address these limitations through multi-site replication studies to establish broader applicability and extended follow-up assessments to evaluate sustained benefits over 6–12 months. Additionally, incorporating behavioral measures of coping effectiveness and objective indicators of legal knowledge application would strengthen the evidence base. Component analysis studies examining the relative effectiveness of micro-learning, narrative presentation, and peer discussion elements would inform intervention optimization and resource allocation for diverse educational contexts.

### Implications for practice and policy

These findings have important implications for legal education policy in Chinese higher education. The demonstrated effectiveness of theory-based multimedia approaches suggests that institutions should integrate such methods into legal publicity programs, particularly given their cost-effectiveness and scalability.

The finding that positive coping strategies fully mediated the intervention’s effect on anxiety suggests that legal education programs should explicitly incorporate coping skill development rather than focusing solely on knowledge transmission. Additionally, the intervention’s consistent effectiveness across demographic subgroups supports universal implementation strategies without requiring costly demographic-specific adaptations.

The intervention’s success through existing institutional structures demonstrates that educational innovation need not require extensive new resources. Strategic enhancement of familiar platforms and established procedures can yield substantial improvements in both educational and psychological outcomes, making this approach suitable for widespread implementation in resource-constrained educational environments. These results also highlight opportunities for interdisciplinary collaboration between legal education, student affairs, and mental health professionals.

## Conclusion

This cluster randomized controlled trial demonstrates that theory-based interactive multimedia legal publicity effectively reduces anxiety and enhances adaptive coping among Chinese vocational college students. The integration of micro-learning principles, narrative psychology, and social cognitive theory created accessible learning experiences that addressed both cognitive and emotional dimensions of legal awareness. Mediation analysis revealed that positive coping strategies fully mediated the intervention-anxiety relationship, demonstrating that the intervention reduces anxiety primarily by enhancing adaptive coping capabilities. The intervention’s consistent effectiveness across demographic subgroups and its reliance on familiar platforms and existing institutional structures support feasible widespread implementation. While limitations include the single-institution design and lack of long-term follow-up, these findings contribute to understanding the psychological mechanisms of multimedia educational interventions and support integration of coping skill development within legal education programming.

## Data Availability

The raw data supporting the conclusions of this article will be made available by the authors, without undue reservation.

## References

[ref1] Bantjes J. Hunt X. Cuijpers P. Kazdin A. E. Kennedy C. J. Luedtke A. . (2024). Comparative effectiveness of remote digital gamified and group CBT skills training interventions for anxiety and depression among college students: results of a three-arm randomised controlled trial. Behav. Res. Ther. 178:104554. doi: 10.1016/j.brat.2024.104554, 38714104

[ref2] Bennett M. Christensen K. (2024). Use of virtual reality (VR) as a clinical tool for management of self-perceived anxiety in college students. J. High. Educ. Theory Pract. 24, 58–67. doi: 10.33423/jhetp.v24i1.6761

[ref3] Chen Y. Zhang Y. Yu G. (2022). Prevalence of mental health problems among college students in mainland China from 2010 to 2020: a meta-analysis. Adv. Psychol. Sci. 30, 991–1004. doi: 10.3724/Sp.J.1042.2022.00991

[ref4] Cuijpers P. Cristea I. A. Karyotaki E. Reijnders M. Huibers M. J. (2016). How effective are cognitive behavior therapies for major depression and anxiety disorders? A meta-analytic update of the evidence. World Psychiatry 15, 245–258. doi: 10.1002/wps.20346, 27717254 PMC5032489

[ref5] Fu H. Pan M. Lai M. (2024). Sources of negative emotions and tactics of self-emotion regulation among college students during Covid-19 school closure in China. Front. Public Health 12:1265350. doi: 10.3389/fpubh.2024.1265350, 38572013 PMC10987727

[ref6] Gabrielli S. Rizzi S. Bassi G. Carbone S. Maimone R. Marchesoni M. . (2021). Engagement and effectiveness of a healthy-coping intervention via Chatbot for university students during the Covid-19 pandemic: mixed methods proof-of-concept study. JMIR Mhealth Uhealth 9:e27965. doi: 10.2196/27965, 33950849 PMC8166265

[ref7] Ge P. Tan C. Liu J. Liu J.-X. Cai Q. Zhao S.-Q. . (2024). Prevalence of subthreshold depression and its related factors in Chinese college students: a cross-sectional study. Heliyon 10:e32595. doi: 10.1016/j.heliyon.2024.e32595, 38988518 PMC11233893

[ref8] Hedges L. V. Hedberg E. C. (2007). Intraclass correlation values for planning group-randomized trials in education. Educ. Eval. Policy Anal. 29, 60–87. doi: 10.3102/0162373707299706

[ref9] Hofstede G. (2011). Dimensionalizing cultures: the Hofstede model in context. Online Read. Psychol. Cult. 2:8. doi: 10.9707/2307-0919.1014

[ref10] Hyde J. S. (2014). Gender similarities and differences. Annu. Rev. Psychol. 65, 373–398. doi: 10.1146/annurev-psych-010213-115057, 23808917

[ref11] Kulkarni S Weir K. (2024) “Translating cognitive behavioural therapy strategies through VR interactions to tackle anxiety symptoms in university students.” In: IEEE gaming, entertainment, and media conference (GEM). New York, NY: IEEE

[ref12] Lattie E. G. Adkins E. C. Winquist N. Stiles-Shields C. Wafford Q. E. Graham A. K. (2019). Digital mental health interventions for depression, anxiety, and enhancement of psychological well-being among college students: systematic review. J. Med. Internet Res. 21:e12869. doi: 10.2196/12869, 31333198 PMC6681642

[ref13] Laure T. Remmerswaal D. Konigorski S. Engels R. C. M. E. Boffo M. (2025). Optimization of a mobile transdiagnostic emotion regulation intervention for university students: a micro-randomized trial. Stress. Health 41:e3507. doi: 10.1002/smi.3507, 39707816 PMC11662259

[ref14] Li J. (2012). Cultural foundations of learning: east and west. Cambridge: Cambridge University Press.

[ref15] Luo W. Mohammed J. (2023). Mental health status and coping strategies of Chinese university students during the Covid-19 pandemic: a rapid review. PLoS One 18:e0296309. doi: 10.1371/journal.pone.0296309, 38134210 PMC10745188

[ref16] Man S. S. Li X. Lin X. J. Lee Y.-C. Chan A. H. S. (2024). Assessing the effectiveness of virtual reality interventions on anxiety, stress, and negative emotions in college students: a meta-analysis of randomized controlled trials. Int. J. Hum. Comput. Interac. 41, 10495–10511. doi: 10.1080/10447318.2024.2434957

[ref17] Pan Q. Fu W. Zhang Y. (2024). Exploring the relationship between coping styles and well-being among Chinese university students: a longitudinal study based on the transactional stress model. Appl. Psychol. Health Well Being 16, 1584–1605. doi: 10.1111/aphw.12543, 38622051

[ref18] Rathod S. Gega L. Degnan A. Pikard J. Khan T. Husain N. . (2018). The current status of culturally adapted mental health interventions: a practice-focused review of meta-analyses. Neuropsychiatr. Dis. Treat. 14, 165–178. doi: 10.2147/Ndt.S138430, 29379289 PMC5757988

[ref19] Shi W. Zhang G. (2024). Psychological disorders and health-promoting lifestyle among Chinese college students: a comprehensive exploration. J. Infrastruct. Policy Dev. 8:14. doi: 10.24294/jipd8485

[ref20] Tan C. (2017). Confucianism and education. Oxford: Oxford Research Encyclopedia of Education.

[ref21] Tian L. Zhang Q. Tsai C.-L. Owusu G. (2025). Coping profiles among Chinese college students: associations with depression and life satisfaction. Counsel. Psychol. 53, 150–173. doi: 10.1177/00110000241312982

[ref22] Xie Y. (1998). Reliability and validity of the simplified coping style questionnaire. Chin. J. Clin. Psychol. 6, 114–115.

[ref23] Xu H. Xue R. Hao S. (2024). Investigation of the psychological health status and influencing factors of vocational college students in Beijing. J. Educ. Res. Rev. 12, 25–36. doi: 10.33495/jerr_v12i2.23.144

[ref24] Yang J. Song X. Zhang J. Zheng Y. Chen G. Bu T. . (2024). Serial multiple mediating role of coping style and anxiety in the relationship between life events and academic satisfaction in Chinese medical undergraduates. Front. Public Health 12:1427616. doi: 10.3389/fpubh.2024.1427616, 39651481 PMC11621852

[ref25] Zulhemay M. N. Kushan A. L. Nasaruddin N. I. S. (2024). “Anxiscape”: an adventure in conquering anxiety via game-based learning for students. Inf. Manag. Bus. Rev. 16, 49–54. doi: 10.22610/imbr.v16i4(S)I.4276

[ref26] Zung W. W. (1971). A rating instrument for anxiety disorders. Psychosomatics 12, 371–379. doi: 10.1016/S0033-3182(71)71479-0, 5172928

